# Development and Evaluation of the Biological Adjuvant Bivalent Vaccine for Preventing Newcastle Disease and Infectious Bursal Disease

**DOI:** 10.1155/2023/5904280

**Published:** 2023-06-28

**Authors:** Xiaochen Guo, Wenying Sun, Changhui Hu, Xiangxiang Wang, Shuang Li, Ruihan Qin, Yixuan Wang, Jinmiao Liu, Zhiqiang Xue, Huijuan Li, Tingting Zhang, Wei Wang, Yunpeng Guo, Jiechao Yin, Ming Jiang, Han Zhao, Lan Wei, Jingmin Zhang, Guiping Ren

**Affiliations:** ^1^Biopharmaceutical Lab, College of Life Science, Northeast Agricultural University, Harbin 150030, China; ^2^Institute of Microbiology, Heilongjiang Academy of Sciences, Harbin 150010, China; ^3^Research Center of Genetic Engineering of Pharmaceuticals of Heilongjiang Province, Northeast Agricultural University, Harbin 150030, China; ^4^Key Laboratory of Agricultural Biological Functional Gene, Northeast Agricultural University, Harbin 150030, China

## Abstract

Newcastle disease (ND) and infectious bursal disease (IBD) are both one of the most economically important infectious diseases, which cause high infection rate and mortality of chickens and restrict the development of the poultry industry. Currently, vaccination is the most effective method to prevent ND and IBD. In this study, the biological adjuvant bivalent vaccine (rClone30-VP2L-chGM-CSF) was developed for preventing ND and IBD. Furthermore, the immune and protective effects of rClone30-VP2L-chGM-CSF were evaluated in specific-pathogen-free chickens. The experimental results indicated that chickens immunized with rClone30-VP2L-chGM-CSF vaccine significantly improved anti-IBDV and anti-NDV antibody titer; the level of CD4^+^ T, CD8^+^ T, B^+^, MHC-II^+^, and monocyte/macrophagocyte+ cells; the concentrations of IL-1*β*, IL-4, IL-17, IFN-*α*, IFN-*β*, and IFN-*γ*; and the splenocyte proliferation rate. Anti-NDV antibody titer reached the theoretical protection value of 4log2 at 7 days after immunization in chickens of rClone30-VP2L-chGM-CSF group; however, which at 14 days after immunization in chickens of rClone30-VP2L and rClone30. These results showed that chickens immunized with rClone30-VP2L-chGM-CSF stimulated stronger immune response than those with the rClone30-VP2L and rClone30. Chickens were challenged with virulent IBDV BC6/85, which were protected in the rClone30-VP2L-chGM-CSF group. Furthermore, IBDV RNA was not measured, and there appeared to be little apoptosis in the bursa of Fabricius in the rClone30-VP2L-chGM-CSF group. Therefore, rClone30-VP2L-chGM-CSF is a promising vaccine candidate against infectious bursal disease virus (IBDV) and newcastle disease virus (NDV), and it provides an idea for developing other poultry vaccines.

## 1. Introduction

Infectious bursal disease (IBD) is caused by infectious bursal disease virus (IBDV), and it is an acute, highly contagious, and viral infectious disease [1, 2]. IBDV causes bursal atrophy and immunosuppression in young chickens and major economic losses in the poultry industry worldwide [3]. The serotypes of IBDV contain serotypes I and II. Serotype I contains the pathogenic strains, whereas serotype II strains mainly isolate from turkeys, which cause neither disease nor protection against serotype I strains in chickens. The pathogenic serotype I field isolates can be grouped into classical virulent or very virulent pathotypes and antigenic variant strains [4]. IBDV is very stable in the environment, even after the chicken houses were disinfected thoroughly, it still exists. At present, vaccines contain inactivated, live, and subunit vaccines for preventing IBD [4–6]. The live vaccines contain low virulent and moderate virulent vaccines. The immune effect of low virulent vaccines was poor against the maternal antibody and very virulent IBDV. Moderate virulent vaccines cause lesions in the bursa of Fabricius (BF) and immunosuppression, which reduces the reactivity to chickens for other vaccines [7]. Though the inactivated vaccine was relatively safe, it usually shows relatively weak immunogenicity and low protective effect. In addition, the live and inactivated vaccines include the intact pathogenic microorganism, which may lead to the spread of pathogenic microorganisms. With the development of gene engineering technique, genetic engineering vaccines are developed and improve the safety of vaccines. VP2 is the principal host-protective antigen of IBDV and induces the generation of neutralizing antibodies [8], while the VP2 neutralizing epitope antigen (VP2L) utilizes a part of the protective antigen VP2 that consists of five neutralizing epitopes, and when it was prepared as a subunit vaccine which could induce to produce higher neutralizing antibodies level and protect chickens from IBDV BC6/85 virulent strain [9]. However, VP2L antigen as a subunit vaccine need to be expressed and purified, it is time-consuming, labor-intensive, and improves the production cost of vaccines. Hence, it is essential to prepare a safe and effective viral vector genetic engineering vaccine with the VP2L gene for preventing IBD.

Newcastle disease (ND) is caused by Newcastle disease virus (NDV) that is an enveloped, nonsegmented, negative-stranded RNA virus. ND causes high infection rate and mortality of chickens, becoming one of the important factors for restricting the development of the poultry industry [10]. The NDV genome is approximately 15.2 kb and contains six transcriptional units, encoding the nucleocapsid protein (NP), phosphoprotein (P), matrix protein (M), fusion protein (F), hemagglutinin-neuraminidase protein (HN), and large polymerase protein (L) [11]. Since reverse genetics technology was first applied to rescue infectious NDV from recombinant cDNA in 1999 [12], many NDV clones have been prepared and are used as vectors to express foreign genes for vaccines. The bivalent vaccines are developed to prevent infectious viral diseases rapidly because the bivalent vaccines are more time-saving and labor-saving and the production cost is lower. Therefore, it is essential to prepare a safe and effective biological adjuvant bivalent vaccine as the genetic engineering vaccine for preventing ND and IBD.

Granulocyte-macrophage colony-stimulating factor (GM-CSF) is a biology adjuvant and has many biological activities. GM-CSF induces antigen presenting cell (APC) to differentiate and maturate and activates neutrophils, macrophages, and dendritic cells, indicating that it plays an important role in immune response [13, 14]. GM-CSF also increased the expression of major histocompatibility complex II (MHC-II) molecules in APCs [15]. So, GM-CSF gene was used to increase the immune efficacy of the viral vector bivalent vaccine.

In this study, the biological adjuvant bivalent vaccine (rClone30-VP2L-chGM-CSF) was obtained with recombinant NDV expressing VP2L and chicken GM-CSF (chGM-CSF). Furthermore, the immune effect of the biological adjuvant bivalent vaccine was evaluated for preventing ND and IBD in specific-pathogen-free (SPF) chickens.

## 2. Materials and Methods

### 2.1. Plasmids, Virus, Antibodies, Cells, Animals, and Primers

pTM-NP, pTM-P, pTM-L, pMD18-T-VP2L, recombinant NDV pBrClone30-chGM-CSF vector, recombinant NDV pBrClone30 vector, rClone30-chGM-CSF virus, rClone30 virus, rabbit anti-VP2 polyclonal antibody, BHK21 cell, and DF1 cell were from our laboratory [16]. The FITC-labeled anti-chicken CD3 antibody, PE-labeled anti-chicken CD8a antibody, PE-labeled anti-chicken CD4 antibody, PE-labeled anti-chicken monocyte/macrophagocyte antibody, PE-labeled anti-chicken MHC-II antibody, and PE-labeled anti-chicken Bu-1 antibody were purchased in the SouthernBiotech, USA. The HRP-labeled anti-rabbit antibody was purchased in the R&D Systems, USA. IBDV BC6/85 strains were purchased from the Chinese National Institute for Supervision of Veterinary Pharmaceuticals. IBDV-B87 strain was purchased in the Harbin Pharmaceutical Group. 9 days SPF chicken embryos and 14-day-old SPF chickens were purchased from the Harbin Veterinary Research Institute, China. All the PCR primers are listed in Table 1.

### 2.2. Recombinant NDV Plasmids Construction and Recombinant Virus Rescue

pMD18-VP2L plasmid was used as the template, and VP2L gene was subcloned with P1 and P2 primers or P3 and P4 primers by PCR, respectively. Then, the PCR products of VP2L were ligated into the vector pBrClone30 using *Sac* II and *Pme* I and named the pBrClone30-VP2L. And PCR products of VP2L were ligated into the vector pBrClone30-chGM-CSF using *Sac* II and named the pBrClone30-VP2L-chGM-CSF. The VP2L gene was inserted between the P gene and M gene in the viral genome.

pTM1-NP, pTM1-P, pTM1-L, and pBrClone30-VP2L-chGM-CSF or pBrClone30-VP2L were cotransfected into 80% confluence of BHK-21 cells in 6-well plates by Lipofectamine 3000 [17]. The supernatant of BHK-21 cells was inoculated into the allantoic cavities of 9-day-old embryonated SPF chicken eggs. The allantoic fluid was harvested and titrated by hemagglutination (HA) test after 72 h. The virus in the HA-positive allantoic fluid was conserved at −80°C. The rescued viruses were named rClone30-VP2L-chGM-CSF and rClone30-VP2L, respectively.

### 2.3. Identification of the Recombinant Viruses by RT-PCR

The VP2L gene of the rClone30-VP2L and rClone30-VP2L-chGM-CSF was identified by RT-PCR. Total RNA was extracted from the recombinant viruses using the virus RNA kit (QIAGEN, Germany) and reverse transcribed to cDNAs using Superscript II (Invitrogen) and random hexamer oligonucleotide primers. Then, VP2L gene was amplified using its own primers by PCR, and the PCR product was sequenced for inserting fidelity.

### 2.4. Measure of Exogenous Gene Expression by Western Blot

The expression of VP2L gene was detected in DF1 cells by Western blot. DF-1 cells were infected with 0.1 MOI chimeric virus and cultured with DMEM medium containing 2% fetal bovine serum (FBS). The supernatants were harvested at 72 h postinfection and detected for the expression of VP2L gene by Western blot [9]. The rClone30 and DMEM medium were used as a negative control.

### 2.5. HA, EID_50_, ICPI, and MDT Assays of the Recombinant Viruses

The titration of recombinant viruses was performed by HA and 50% egg infectious dose (EID_50_). The intracerebral pathogenicity index (ICPI) and the mean death time (MDT) assays were performed with the recombinant viruses according to [18]. NDV rClone30 virus was used as a control. The calculations of the EID_50_, ICPI, and MDT were performed as [18].

### 2.6. The Growth Curve of the Recombinant Viruses

The replication efficiency of recombinant viruses was measured in DF1 cells. Triplicate monolayers of cells were infected with 0.1 MOI chimeric virus and cultured in DMEM medium containing 2% FBS. The supernatants were collected at 24 h, 48 h, 72 h, and 96 h postinfection and measured for TCID_50_ in DF1 cells. The NDV rClone30 was used as the control. The titers were expressed as log10 TCID_50_/mL. The experiments were performed three times.

### 2.7. Transmission Electron Microscopy

The images of recombinant viruses were acquired with negative staining of the sample with 2% (w/v) aqueous uranyl acetate by HITACHI HT7800 microscope. The NDV rClone30 was used as a control.

### 2.8. Animal Immunization

Seventy 14-day-old SPF chickens were randomly divided into rClone30-VP2L-chGM-CSF group, rClone30-VP2L group, rClone30-chGM-CSF group, rClone30 group, IBDV-B87 group, PBS group, and challenge group. Chickens were immunized intramuscularly with 10^6^ EID_50_ doses of recombinant viruses. Chickens were immunized with a commercial IBDV-B87 vaccine via the oculonasal route in the IBDV-B87 group as a vaccine control. Chickens were immunized with PBS and nonchallenged in the PBS group as a negative control group, and chickens were immunized with PBS and challenged in the challenge group as a challenge control. All chickens were immunized in the same way at 28 days of age again (Table 2). The spleen and BF were collected from three chickens 28 days after immunization. Blood samples were obtained at 7, 14, 21, and 28 days after immunization.

### 2.9. Challenge Study

At 28 days after immunization, seven chickens were challenged with 0.1 mL of 100 median bursa infective doses (100 BID_50_) of IBDV BC6/85 by oral route and observed clinically for 96 h, except the PBS group. The BF/body weight (BW) ratio was calculated in all chickens [9]. Part of the BF was stored at −80°C for enzyme-linked immunosorbent assay (ELISA) and real-time PCR analysis, and a portion was used for histopathologic analysis.

### 2.10. Anti-IBDV and Anti-NDV Antibody Titers Detection

Blood samples were obtained and isolated serum at 7, 14, 21, and 28 days after immunization. The anti-IBDV antibody titer was measured by a commercial ELISA kit (IDEXX, USA, Cat: 99-09261). The anti-NDV antibody titer was detected by HI.

### 2.11. Virus Neutralization Assay (VNA)

Virus neutralization assay was detected with the serum samples in DF1 cells according to [9].

### 2.12. Detection of Cytokines in the Serum by ELISA

The cytokines in the serum samples were measured by commercial ELISA kits (chicken IL-4 (Cat: AD0453Ch), IL-17 (Cat: AD0459Ch), IFN-*α* (Cat: AD0472Ch), and IFN-*γ* (Cat: AD0470Ch) ELISA kits were from Andy gene, China). The result was confirmed by standard curves.

### 2.13. FCM Analysis

At 7, 14, 21, and 28 days after immunization, the CD4^+^ T cells, CD8^+^ T cells, B^+^ cells, MHC-II^+^ cells, and monocyte/macrophagocyte^+^ cells were detected with blood samples by FCM. At 28 days after immunization, the CD4^+^ T cells, CD8^+^ T cells, B^+^ cells, and monocyte/macrophagocyte+ cells were detected with the spleen tissue by FCM [9].

### 2.14. Analysis of the mRNA Levels of Cytokines by Real-Time PCR

Cytokines IL-1*β*, IL-4, IL-17, IFN-*α*, IFN-*β*, and IFN-*γ* were detected with white blood cells and the spleen tissue by real-time PCR [9]. These primers are shown in Table 3.

### 2.15. Splenocyte Proliferation Assay

The spleen was aseptically collected from three chickens at 28 days after immunization. Splenocyte proliferation assay were detected according to [9]. Splenocytes were stimulated with NDV rClone30, IBDV-B87, VP2 antigen, con A, or 1640 medium.

### 2.16. Analysis of the Viral Load in the BF by Real-Time PCR

The BFs were harvested at 96 h postchallenge, and the viral load was detected by real-time PCR according to [9].

### 2.17. Histopathological Study

The BFs were obtained at 96 h postchallenge, which were stained with hematoxylin and eosin. The pathological sections were assessed and recorded the bursal lesion scores according to [19].

### 2.18. Analysis of Apoptotic-Related Proteins in the BFs by ELISA

The Bcl-2 and Bax proteins were measured in the BFs by commercial ELISA kits (chicken Bcl-2 (Cat: AD0534Ch) and Bax (Cat: AD0516Ch) ELISA kits are from Andy gene, China). The results were confirmed by standard curves.

### 2.19. Statistical Analysis

All data are expressed as mean ± S.D of at least three independent experiments. Statistically significant differences were analyzed by one-way or two-way ANOVA between two groups using GraphPad Prism 5.0 software. Data were considered as significant differences at *P* < 0.05.

## 3. Results

### 3.1. Rescue of Recombinant Viruses

**Figure 1:**
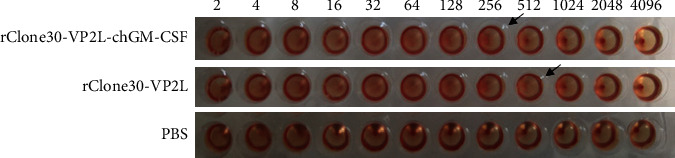
The rescue of the recombinant viruses. The titration of rClone30-VP2L-chGM-CSF and rClone30-VP2L was performed by HA.

The recombinant pBrClone30-VP2L-chGM-CSF and pBrClone30-VP2L were constructed successfully (data not shown). Then, they were rescued by a reverse genetic operating system. The HA of rClone30-VP2L-chGM-CSF and rClone30-VP2L was 2^8^ and 2^9^, respectively (Figure 1). The result showed the recombinant viruses were obtained successfully.

### 3.2. Identification of Recombinant Viruses

**Figure 2:**
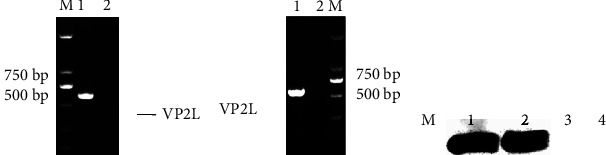
The identification of the recombinant viruses: (a) the identification of rClone30-VP2L-chGM-CSF virus by RT-PCR. Lane M: DL2000 marker, lane 1: rClone30-VP2L-chGM-CSF virus, and lane 2: negative control. (b) The identification of rClone30-VP2L virus by RT-PCR. Lane M: DL2000 marker, lane 1: rClone30-VP2L virus, and lane 2: negative control. (c) The identification of the recombinant viruses by Western blot. Lane M: prestained protein marker, lane 1: rClone30-VP2L-chGM-CSF virus, lane 2: rClone30-VP2L virus, lane 3: rClone30 virus, and lane 4: DMEM medium.

The VP2L gene was amplified from the viral genome by RT-PCR and confirmed by DNA sequencing. The results showed that the amplified target fragment was the same size as the expected fragment (Figures 2(a) and 2(b)), and the identity of the nucleotide sequence was 100% by DNAMAN comparison, which indicated that the VP2L gene was successfully inserted into the rClone30 and rClone30-chGM-CSF vectors.

The expression of VP2L gene was measured in DF1 cells infected with the recombinant virus by Western blot. The results indicated that VP2L gene was successfully expressed in the recombinant virus infected cells (Figure 2(c)).

### 3.3. The Virus Titer and Pathogenicity Indexes of Recombinant Viruses

The virus titer and the pathogenicity assay of recombinant viruses were measured by HA, EID_50_, ICPI, and MDT assays. The results are presented in Table 4. The results of the HA test showed that the HA titer of rClone30-VP2L-chGM-CSF was 2^8^, that of rClone30-VP2L was 2^9^, and that of rClone30 was 2^9^. The EID_50_ for the rClone30-VP2L-chGM-CSF, rClone30-VP2L, and rClone30 were 10^7.6^, 10^8^, and 10^8^, respectively. The MDT observed for the rClone30-VP2L-chGM-CSF, rClone30-VP2L, and rClone30 was greater than 120 h. The results of ICPI for rClone30-VP2L-chGM-CSF, rClone30-VP2L, and rClone30 were 0.00. There was no significant difference between the recombinant viruses and rClone30 in terms of pathogenicity.

### 3.4. Growth Characteristics of Recombinant Viruses

**Figure 3:**
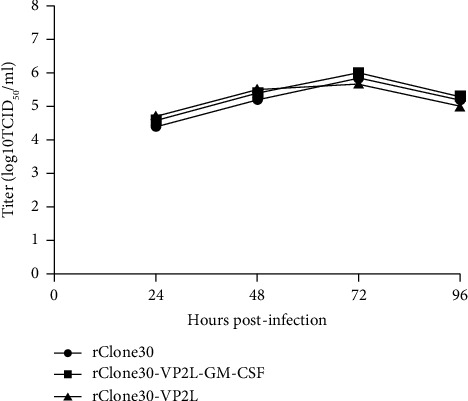
The growth curve of recombinant viruses. DF1 cells were infected with viruses at 0.1 MOI and supernatants were harvested after 24 h, 48 h, 72 h, and 96 h for TCID_50_ calculation. Values represent the means of three independent experiments.

The growth characteristics of recombinant viruses were analyzed in DF1 cells, and rClone30 was used as a control. The result showed that the insertion of the VP2L gene did not influence the growth kinetics of rClone30 (Figure 3).

### 3.5. Transmission Electron Microscopy of Recombinant Viruses

**Figure 4:**
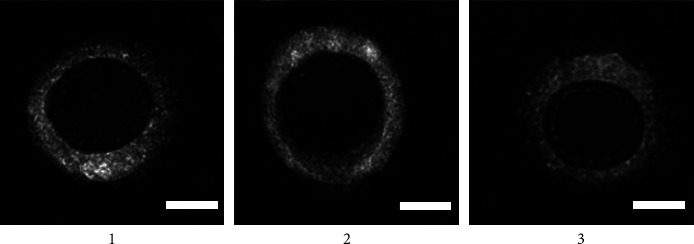
Transmission electron microscopy of recombinant viruses. Lane 1: rClone30-VP2L-chGM-CSF virus; lane 2: rClone30-VP2L virus; lane 3: rClone30 virus, bar = 200 nm.

The images of recombinant viruses were acquired by the HITACHI HT7800 microscope. The results indicated that rClone30-VP2L-chGM-CSF, rClone30-VP2L, and rClone30 virions were mostly spherical or elliptical. The diameter of the virions was about 100–400 nm, and there were peripheral capsule membranes embedded with fibrines. The results showed that rClone30-VP2L-chGM CSF and rClone30-VP2L had no changes in morphological and structural characteristics with rClone30 (Figure 4).

### 3.6. Detection of Anti-IBDV and Anti-NDV Antibody Titer

**Figure 5:**
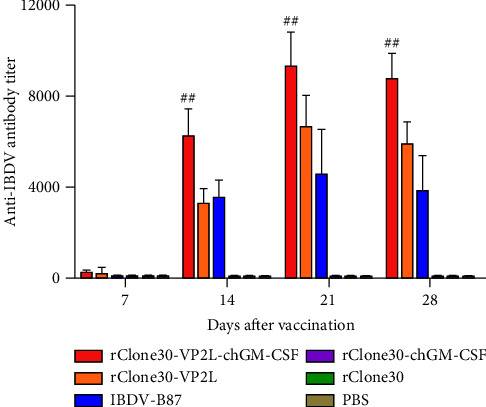
Measurement of anti-IBDV antibody titer by ELISA at 7, 14, 21, and 28 days after immunization. Data were analyzed and expressed as mean ± SD, ^##^*P* < 0.01 vs rClone30-VP2L-treated chickens.

At 7, 14, 21, and 28 days after immunization, anti-IBDV antibody titer was detected using an ELISA kit. The result suggested that the antibody titer elevated from 7 to 21 days and descended at 28 days after immunization. From 14 to 28 days postimmunization, the antibody titer of chickens in the rClone30-VP2L-chGM-CSF group was significantly higher than those in the rClone30-VP2L group (^##^*P* < 0.01) and the IBDV-B87 group (Figure 5).

**Figure 6:**
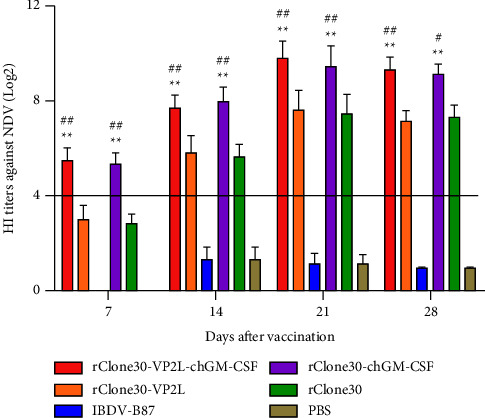
Detection of anti-NDV antibody titer by HI at 7, 14, 21, and 28 days after immunization. Data were analyzed and expressed as mean ± SD, ^#^*P* < 0.05, ^##^*P* < 0.01 vs rClone30-VP2L-treated chickens and expressed as mean ± SD, ^∗∗^*P* < 0.01 vs rClone30-treated chickens.

Anti-NDV antibody titer was assessed by HI assay at 7, 14, 21, and 28 days after immunization. The result indicated that the HI antibody titer was on the rise at 7 to 21 days and descended at 28 days after immunization. At 7 days after immunization, the HI antibody titer of chickens in rClone30-VP2L-chGM-CSF and rClone30-chGM-CSF groups was higher than the protection critical value of 4 log2; however, that in rClone30-VP2L and rClone30 groups it did not reach the protection critical value of 4 log2. The HI antibody titer of chickens in rClone30-VP2L-chGM-CSF and rClone30-chGM-CSF groups was dramatically higher than those in rClone30-VP2L and rClone30 groups from 7 to 28 days after immunization (^*∗∗*^*P* < 0.01, ^#^*P* < 0.05, or ^##^*P* < 0.01) (Figure 6).

### 3.7. Measurement of Anti-IBDV Neutralizing Antibody Titer

**Figure 7:**
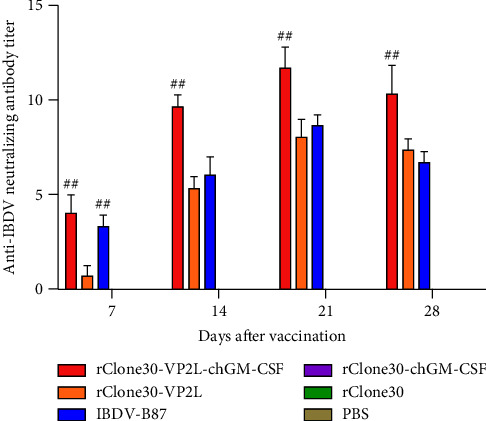
Measurement of anti-IBDV neutralizing antibody titer at 7, 14, 21, and 28 days after immunization. Data were analyzed and expressed as mean ± SD, ^##^*P* < 0.01 vs rClone30-VP2L-treated chickens.

The neutralizing antibody titer was measured using VNA. The result showed that the neutralizing antibody titer elevated from 7 to 21 days and descended at 28 days after immunization. From 7 to 28 days postimmunization, the neutralizing antibody titer of chickens in rClone30-VP2L-chGM-CSF group was significantly higher than those in the rClone30-VP2L group (^##^*P* < 0.01) and the IBDV-B87 group (Figure 7).

### 3.8. Detection of Inflammatory Cytokines in the Serum

**Figure 8:**
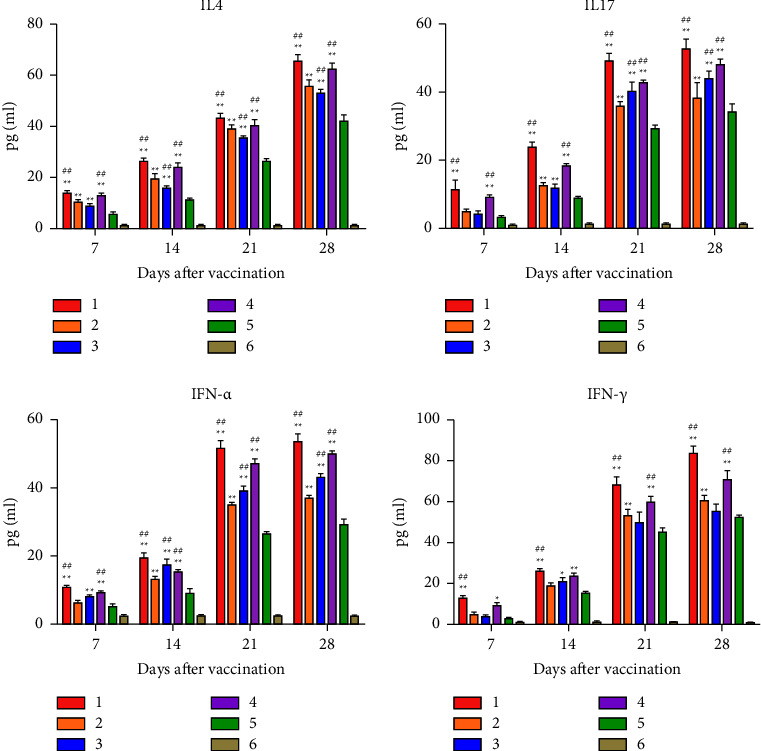
Measurement of inflammatory cytokines in the serum. Detection of IL-4, IL-17, IFN-*α*, and IFN-*γ* in the serum at 7, 14, 21, and 28 days after immunization by ELISA. Lane 1: rClone30-VP2L-chGM-CSF, lane 2: rClone30-VP2L, lane 3: IBDV-B87, lane 4: rClone30-chGM-CSF, lane 5: rClone30 and lane 6: PBS. Data were analyzed and expressed as mean ± SD; ^#^*P* < 0.05 and ^##^*P* < 0.01 vs. rClone30-VP2L-treated chickens and expressed as mean ± SD; ^*∗*^*P* < 0.05 and ^*∗∗*^*P* < 0.01 vs. rClone30-treated chickens.

The concentrations of inflammatory cytokines (IL-4, IL-17, IFN-*α*, and IFN-*γ*) were analyzed in the serum by ELISA at 7, 14, 21, and 28 days after immunization. The results showed that the concentration of inflammatory cytokines increased from 7 to 28 days after immunization. After immunization, the concentrations of all inflammatory cytokines in chickens of the rClone30-VP2L-chGM-CSF group were significantly higher than those in rClone30-VP2L and rClone30 groups and were higher than those in rClone30-chGM-CSF and IBDV-B87 groups (^*∗∗*^*P* < 0.01 or ^##^*P* < 0.01) (Figure 8).

### 3.9. Immunocyte-Mediated Immune Responses

**Figure 9:**
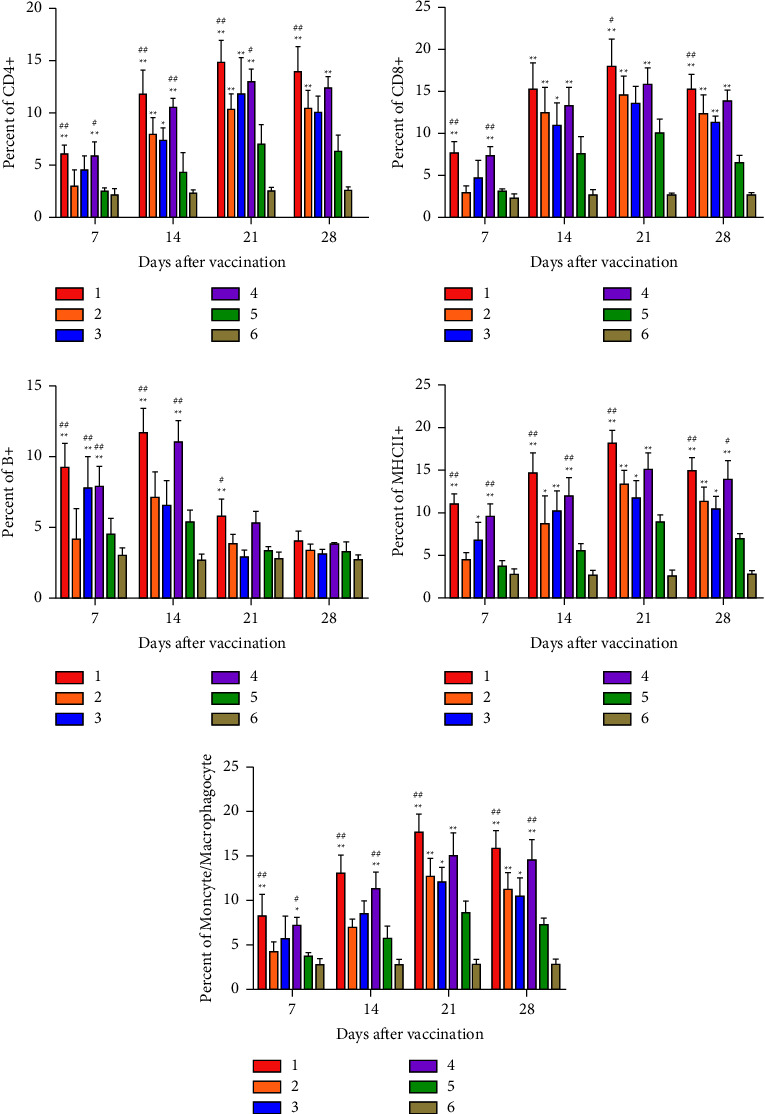
Detection of immunocyte-mediated immune response in white cells. The percentages of CD4^+^ T, CD8^+^ T, B^+^, MHC-II^+^, or monocyte/macrophagocyte cells were detected by FCM at 7, 14, 21, and 28 days after immunization. Lane 1: rClone30-VP2L-chGM-CSF, lane 2: rClone30-VP2L, lane 3: IBDV-B87, lane 4: rClone30-chGM-CSF, lane 5: rClone30, and lane 6: PBS. Data were analyzed and expressed as mean ± SD; ^#^*P* < 0.05 and ^##^*P* < 0.01 vs. rClone30-VP2L-treated chickens and expressed as mean ± SD; ^*∗*^*P* < 0.05 and ^*∗∗*^*P* < 0.01 vs. rClone30-treated chickens.

At 7, 14, 21, and 28 days after immunization, CD4^+^ T, CD8^+^ T, B^+^, MHC-II^+^, and monocyte/macrophagocyte^+^ cells were measured in white blood cells by FCM. The results demonstrated that the percentages of CD4^+^ T, CD8^+^ T, MHC-II^+^, and monocyte/macrophagocyte^+^ cells elevated from 7 to 21 days and descended at 28 days after immunization, while the percentage of B^+^ cells attained its peak at 14 days and descended at 21 days after immunization. After immunization, the percentages of all immune cells of chickens in rClone30-VP2L-chGM-CSF and rClone30-chGM-CSF groups were markedly higher than those in the rClone30 group (^*∗∗*^*P* < 0.01, ^*∗*^*P* < 0.05, ^#^*P* < 0.05, or ^##^*P* < 0.01) and were higher than those in rClone30-VP2L and IBDV-B87 groups (Figure 9).

**Figure 10:**
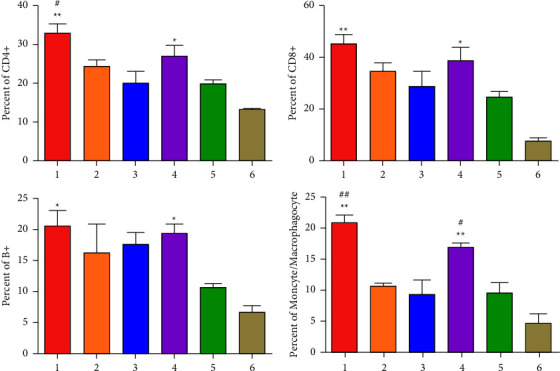
Measurement of immunocyte-mediated immune response in the spleen. The percentages of CD4^+^ T, CD8^+^ T, B^+^, or monocyte/macrophagocyte cells were detected by FCM at 28 days after immunization. Lane 1: rClone30-VP2L-chGM-CSF, lane 2: rClone30-VP2L, lane 3: IBDV-B87, lane 4: rClone30-chGM-CSF, lane 5: rClone30, and lane 6: PBS. Data were analyzed and expressed as mean ± SD; ^#^*P* < 0.05 and ^##^*P* < 0.01 vs. rClone30-VP2L-treated chickens and expressed as mean ± SD; ^*∗*^*P* < 0.05 and ^*∗∗*^*P* < 0.01 vs. rClone30-treated chickens.

At 28 days after immunization, CD4^+^ T, CD8^+^ T, B^+^, or monocyte/macrophagocyte^+^ cells were determined in the spleen by FCM. The results indicated that the percentages of all immune cells of chickens in rClone30-VP2L-chGM-CSF and rClone30-chGM-CSF groups were significantly higher than those in the rClone30 group (^*∗∗*^*P* < 0.01, ^*∗*^*P* < 0.05, ^#^*P* < 0.05, or ^##^*P* < 0.01) and were higher than those in rClone30-VP2L and IBDV-B87 groups in the spleen (Figure 10).

### 3.10. Inflammatory Responses

**Figure 11:**
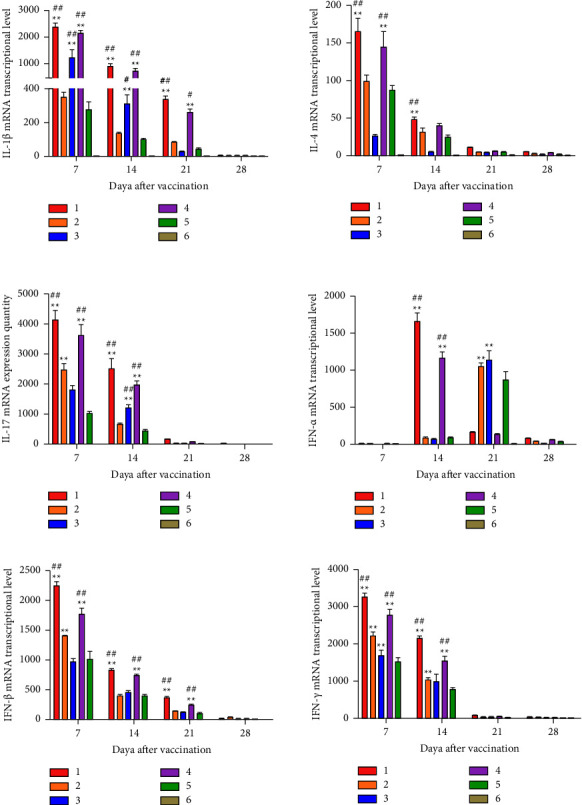
The mRNA expression level of inflammatory cytokines in white cells. Inflammatory cytokines IL-1*β*, IL-4, IL-17, IFN-*α*, IFN-*β*, and IFN-*γ* were analyzed in the white cells by real-time PCR at 7, 14, 21, and 28 days after immunization. Lane 1: rClone30-VP2L-chGM-CSF; Lane 2: rClone30-VP2L; Lane 3: IBDV-B87; Lane 4: rClone30-chGM-CSF; Lane 5: rClone30; Lane 6: PBS. Data were analyzed and expressed as mean ± SD, ^#^*P* < 0.05, ^##^*P* < 0.01 vs rClone30-VP2L-treated chickens and expressed as mean ± SD, ^∗∗^*P* < 0.01 vs rClone30-treated chickens.

At 7, 14, 21, and 28 days after immunization, inflammatory cytokines IL-1*β*, IL-4, IL-17, IFN-*α*, IFN-*β*, and IFN-*γ* were detected by real-time PCR in white cells. mRNA levels of IL-1*β*, IL-4, IL-17, IFN-*β*, and IFN-*γ* showed upregulation at 7 days and downregulation at 14 days after immunization. However, mRNA levels of IFN-*α* of chickens in rClone30-VP2L-chGM-CSF and rClone30-chGM-CSF groups had a rapid upregulation at 14 days, and that in rClone30-VP2L and rClone30 groups it had upregulation at 21 days after immunization. At 7 or 14 days after immunization, mRNA levels of all inflammatory cytokines of chickens in rClone30-VP2L-chGM-CSF and rClone30-chGM-CSF groups were significantly higher than those in the rClone30 group (^*∗∗*^*P* < 0.01, ^#^*P* < 0.05, or ^##^*P* < 0.01) and were higher than those in rClone30-VP2L and IBDV-B87 groups (Figure 11).

**Figure 12:**
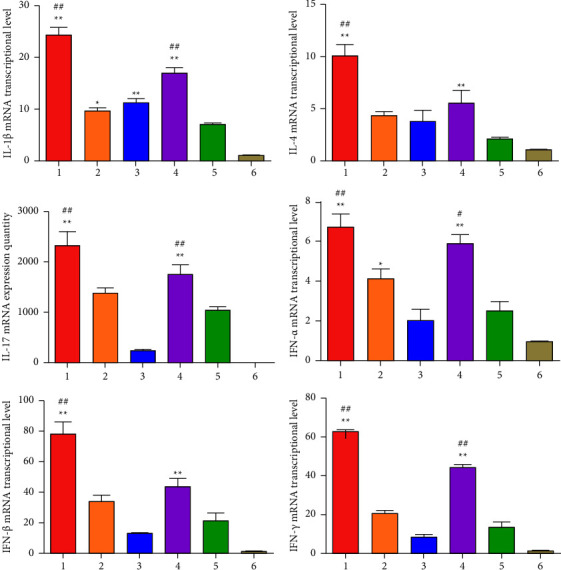
The mRNA expression level of inflammatory cytokines in the spleen. Inflammatory cytokines IL-1*β*, IL-4, IL-17, IFN-*α*, IFN-*β*, and IFN-*γ* were analyzed in the spleen by real-time PCR at 28 days after immunization. Lane 1: rClone30-VP2L-chGM-CSF, lane 2: rClone30-VP2L, lane 3: IBDV-B87, lane 4: rClone30-chGM-CSF, lane 5: rClone30, and lane 6: PBS. Data were analyzed and expressed as mean ± SD; ^#^*P* < 0.05 and ^##^*P* < 0.01 vs. rClone30-VP2L-treated chickens and expressed as mean ± SD; ^*∗*^*P* < 0.05 and ^*∗∗*^*P* < 0.01 vs. rClone30-treated chickens.

At 28 days after immunization, inflammatory cytokines IL-1*β*, IL-4, IL-17, IFN-*α*, IFN-*β*, and IFN-*γ* were measured by real-time PCR in the spleen. The results demonstrated that the mRNA levels of all inflammatory cytokines in chickens of rClone30-VP2L-chGM-CSF and rClone30-chGM-CSF groups were significantly higher than those in the rClone30 group (^*∗∗*^*P* < 0.01, ^*∗*^*P* < 0.05, ^#^*P* < 0.05, or ^##^*P* < 0.01) and were higher than those in rClone30-VP2L and IBDV-B87 groups (Figure 12).

### 3.11. Proliferation of Splenocytes

**Figure 13:**
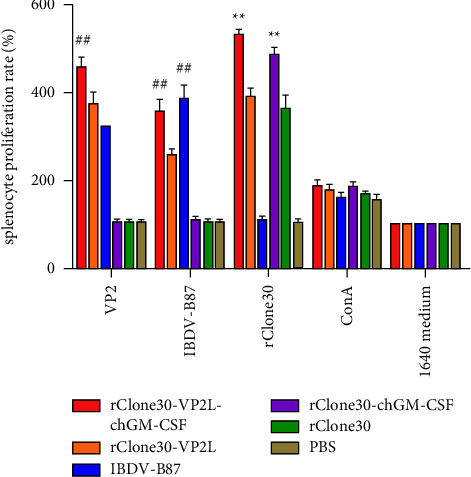
Proliferation of splenocytes. Splenocytes were stimulated with VP2 protein, IBDV-B87, or rClone30 virus in all groups. Con A was used as positive control, and 1640 medium alone was used as negative control. The cell proliferation was measured by CCK-8 assay. Data were analyzed and expressed as mean ± SD, ^##^*P* < 0.01 vs rClone30-VP2L-treated chickens and expressed as mean ± SD, ^∗∗^*P* < 0.01 vs rClone30-treated chickens.

Splenocytes were prepared and stimulated with NDV rClone30, IBDV-B87, and VP2 antigen. The results showed that the splenocyte proliferation rate (SPR) of chickens stimulated with rClone30 virus in rClone30-VP2L-chGM-CSF and rClone30-chGM-CSF groups was markedly higher than that in rClone30-VP2L and rClone30 groups (^*∗∗*^*P* < 0.01). The SPR of chickens stimulated with VP2 protein or IBDV-B87 virus in the rClone30-VP2L-chGM-CSF group was observably higher than that in the rClone30-VP2L group (^##^*P* < 0.01) (Figure 13).

### 3.12. Challenge Protection

**Figure 14:**
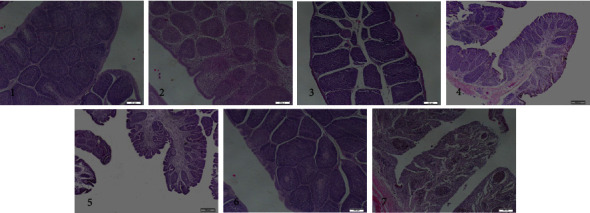
Histopathological of BFs after challenging. The histopathological of BFs in chickens were measured by H&E staining at 7, 14, 21, and 28 days after immunization. Lane 1: rClone30-VP2L-chGM-CSF, lane 2: rClone30-VP2L, lane 3: IBDV-B87, lane 4: rClone30-chGM-CSF, lane 5: rClone30, lane 6: PBS, and lane 7: challenge.

**Figure 15:**
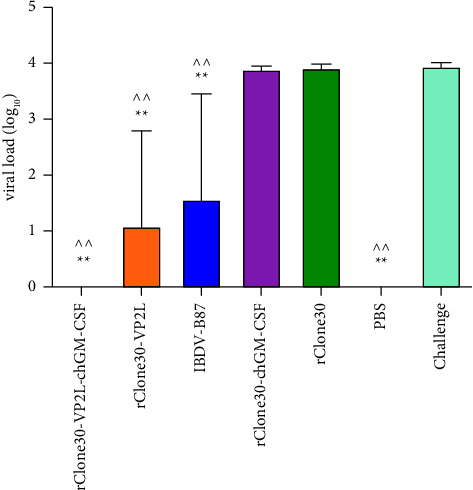
Detection of residual IBDV in the BFs after challenging. The residual IBDV was detected in the BFs of all chickens by real-time PCR. The positive plasmid VP5-T was diluted as log10 dilutions and measured as the standard curve. Data were analyzed and expressed as mean ± SD, ^ ^*P* < 0.01 vs challenge-treated chickens and expressed as mean ± SD, ^∗∗^*P* < 0.01 vs rClone30-treated chickens.

At 28 days after immunization, chickens were challenged with IBDV BC6/85 virus. The bursal lesion scores, BF/BW ratios, IBDV in BF, and protection rate are presented in Table 5. Histopathology was used to evaluate the protective efficacy of these vaccines (Figure 14), and the results indicated that chickens in the rClone30-VP2L-chGM-CSF group had a protection rate of 100%, whereas in the rClone30-VP2L group had a protection rate of 71%. However, in the positive control group, chickens in the IBDV-B87 group had a protection rate of 57%. In addition, residual IBDV in the BFs of all chickens was detected by real-time PCR. The results demonstrated that no IBDV was detected in chickens of the rClone30-VP2L-chGM-CSF group; chickens in the challenge group had a large number of viruses, and the result was consistent with that of the bursal lesion (Figure 15).

**Figure 16:**
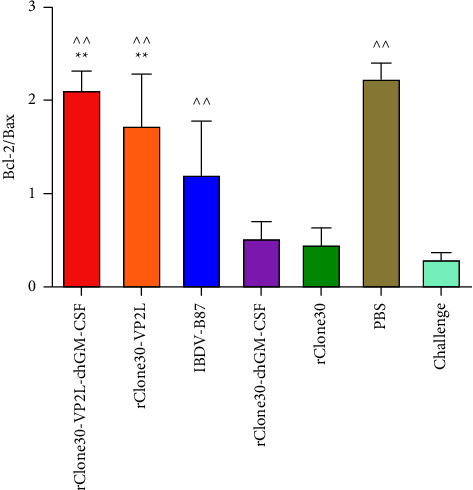
Analysis of apoptosis in the BFs after challenging. Analysis of apoptotic proteins (Bax and Bcl-2) in the BFs by ELISA. Data were analyzed and expressed as mean ± SD, ^ ^*P* < 0.01 vs challenge-treated chickens and expressed as mean ± SD, ^∗∗^*P* ± 0.01 vs rClone30-treated chickens.

The apoptosis-related proteins (Bcl-2 and Bax) were analyzed within the BFs of all chickens. The ratio of Bcl-2/Bax was used to estimate the level of apoptosis. These results demonstrated that there was little apoptosis in chickens of the rClone30-VP2L-chGM-CSF group; however, there was much apoptosis in chickens of the challenge group (Figure 16).

## 4. Discussion

ND was first reported in 1926 and it had a history of more than 90 years, and people had also deeply studied NDV [17]. With the development of reverse genetic manipulation technology, a large number of studies have shown that NDV has been used as a vector to express foreign proteins [20]. NDV can be propagated in chicken embryos and reach a very high titer, so that it greatly reduces the production cost [21]. NDV replicates in the cytoplasm without going through the DNA phase, so there is no possibility of integration with the host cell genome [21]. NDV is relatively stable in genetic, and it can stably express foreign genes and induce humoral and cellular immune responses [21, 22]. In the prevention of ND, a live attenuated vaccine has been widely used and its safety has been fully approved [21]. Therefore, NDV has many advantages as a recombinant virus vector to express foreign proteins.

Vaccines are still the main way to prevent ND. However, the inactivated vaccine and live attenuated vaccine product antibodies slowly, especially the antigen slow release of inactivated vaccine which can stimulate the body to produce antibodies at 14 to 21 days after immunization. The immune empty window is long, so chickens do not have the ability to resist wild virus attacks at the initial stage of immunization. In addition, medium virulent vaccine strains are usually used to prevent IBD; however, it can damage the BF in chickens and lead to immunosuppression, which reduce the reactivity to other vaccines [3]. Therefore, it is very important to develop a biological adjuvant bivalent vaccine that can quickly produce antibodies without harming the BF for preventing IBD and ND.

In general, an intact protective protein of IBDV VP2 is used as viral vector genetic engineering vaccine to prevent IBD; however, in the study, IBDV VP2L gene was inserted in NDV rClone30 recombinant virus vector at P/M site to prevent IBD and ND. The length of VP2 gene was two times higher than that of VP2L gene, so the expression level of VP2L gene may be much higher than that of VP2 gene in the recombinant virus vector. In addition, VP2L protein induces to product higher neutralizing antibodies level than VP2 whole protein. As the GM-CSF gene was also added to NDV rClone30 recombinant virus vector as a biological adjuvant, it can activate macrophages, dendritic cells, and neutrophils; induce APCs differentiation and maturation; and rapidly enhance specific immune response and protective immunity [16, 23]. Therefore, the immune response level of rClone30-VP2L-chGM-CSF could be better than the vaccine that is prepared with recombinant NDV expressing IBDV VP2.

In the study, the recombinant pBrClone30-VP2L-chGM-CSF was constructed successfully, and it was rescued by reverse genetic manipulation and obtained rClone30-VP2L-chGM-CSF virus, and that was able to pass and express foreign genes stably. The MDT and ICPI of recombinant virus rClone30-VP2L-chGM-CSF showed that it was still a weak virus strain without enhancing virulence with its parent strain, so the recombinant virus was safe as a vaccine. The proliferation curve of the recombinant virus showed no difference from that of the parent strain rClone30, indicating that the insertion of foreign genes did not affect the proliferation of the virus. The immune effects of rClone30-VP2L-chGM-CSF were analyzed by an animal experiment. The results indicated that chickens immunized with rClone30-VP2L-chGM-CSF significantly increased the anti-IBDV antibody titer, anti-NDV antibody titer, anti-IBDV neutralizing antibody titer, and levels of B cells compared with the rClone30-VP2L and rClone30. At 7 days after immunization, the B+ cells level of chickens immunized with rClone30-VP2L-chGM-CSF was significantly higher than that with the rClone30-VP2L and rClone30 groups, and anti-NDV antibody titer of chickens immunized with rClone30-VP2L-chGM-CSF reached the theoretical protection value of 4log2; however, which with rClone30-VP2L and rClone30 reached the theoretical protection value at 14 days after immunization, indicating that rClone30-VP2L-chGM-CSF rapidly stimulated stronger humoral immune response. CD4+ helper T cells are divided into two different effector groups, including Th1 and Th2. Th1 mainly enhances the cell-mediated effect, and IFN-*γ* and IL-2 are important cytokine of Th1. Th2 mainly enhances humoral immune function, and the representative factors are IL-4 and IL-10. Interleukin-17 (IL-17) is a newly discovered potent proinflammatory factor, mainly secreted by Th17 cells that can recruit neutrophils and promote the release of inflammatory factors, adhesion molecules, growth factors, and cell proliferation factors by various cells. At 7 days after immunization, chickens immunized with rClone30-VP2L-chGM-CSF observably improved the levels of CD4^+^ T and CD8^+^ T cells and the expression levels of IL-4 and IFN-*γ* compared with rClone30-VP2L and rClone30, indicating that rClone30-VP2L-chGM-CSF rapidly stimulated stronger humoral and cellular immune response, including Th1 and Th2 responses. At 7 days after immunization, chickens immunized with rClone30-VP2L-chGM-CSF markedly improved the levels of monocyte/macrophagocyte^+^ and MHC-II^+^ cells compared with the rClone30-VP2L and rClone30, indicating that rClone30-VP2L-chGM-CSF rapidly stimulated stronger immune response. Compared with chickens immunized with the rClone30-VP2L and rClone30, chickens immunized with rClone30-VP2L-chGM-CSF markedly increased the mRNA expression levels of IL-1*β*, IL-4, IL-17, IFN-*β*, and IFN-*γ* at 7 days after immunization, and that of IFN-*α* at 14 days after immunization. However, the mRNA expression levels of IFN-*α* of chickens in rClone30-VP2L and rClone30 groups reached the peak at 21 days after immunization, indicating that rClone30-VP2L-chGM-CSF rapidly stimulated stronger inflammatory responses, including Th17 response. According to the results of the challenge protection, chickens in the rClone30-VP2L-chGM-CSF group had a protection rate of 100%, and that in rClone30-VP2L and IBDV-B87 groups had a protection rate of 71% and 57%, respectively. In addition, no IBDV was detected in rClone30-VP2L-chGM-CSF and PBS groups, and the result was in accordance with that of the bursal lesion. There were a little apoptosis in rClone30-VP2L-chGM-CSF and PBS groups; however, the bursa existed much apoptosis in the challenge group. These results showed that rClone30-VP2L-chGM-CSF protected chickens from IBDV BC6/85 virulent strain entirely and stimulated stronger humoral and cellular immune response rapidly. In addition, the biological adjuvant bivalent vaccine rClone30-VP2L-chGM-CSF can prevent two diseases, and it simplifies the vaccine preparation process and reduces the production cost; therefore, it is suitable for industrial production.

In summary, the biological adjuvant bivalent vaccine rClone30-VP2L-chGM-CSF prevented IBD and ND and stimulated humoral and cellular immune response rapidly. Therefore, the biological adjuvant bivalent vaccine can prevent IBD and ND as a vaccine candidate, and it provides an idea to develop other vaccines against viral infectious diseases.

## Figures and Tables

**Table 1:** Primers used in the amplification of the target genes.

Primers	Primer sequences (5′-3′)
P1	*CCGCGG*GGGCCACCATGACAAACCTGCAAGATC
P2	*CCGCGG*TTCTACCCGTATTTTTTCTTAATTAGGTGTACTCCCTTGTT
P3	*CCGCGG*GCCACCATGACAAACCTGCAAGATC
P4	*GTTTAAAC*CTTCTACCCGTATTTTTTCTTAATTAGGTGTACTCCCTTGTT

Note. Italic parts are the recognition sites of restriction endonuclease *Sac*II and *Pme*I. P1–P4 are used for cloning the VP2 neutralizing epitope antigen (VP2L) gene.

**Table 2:** Experimental design for chicken immunization.

Groups of vaccine	Administration	Dose (*μ*l)	Number of chickens	Age (days) at vaccination		Days of challenged
				1st	2nd	
rClone30-VP2L-chGM-CSF	Intramuscularly	200	10	14	28	42
rClone30-VP2L	Intramuscularly	200	10	14	28	42
IBDV-B87 vaccine	Oculonasal route	200	10	14	28	42
rClone30-chGM-CSF	Intramuscularly	200	10	14	28	42
rClone30	Intramuscularly	200	10	14	28	42
PBS (negative control)	Intramuscularly	200	10	14	28	
PBS (challenge control)	Intramuscularly	200	10	14	28	42

**Table 3:** Primers used in the real time PCR.

Primers	Primer sequences (5′-3′)
chIL-1*β*F	TGGGCATCAAGGGCTACA
chIL-1*β*R	TCGGGTTGGTTGGTGATG
chIL-4F	AACATGCGTCAGCTCCTGAAT
chIL-4R	TCTGCTAGGAACTTCTCCATTGAA
chIL-17F	CTCCTCTGTTCAGACCACTGC
chIL-17R	ATCCAGCATCTGCTTTCTTGA
chIFN-*α*F	GATTTCGAGCAGGAGATGGCCACAG
chIFN-*α*R	GATCCACATCTGCTGGAAGGTGGAC
chIFN-*β*F	AACGCTCACCTCAGCATCAA
chIFN-*β*R	GTGTCGGAAGCAGTCACGAG
chIFN-*γ*F	CAAAGCCGCACATCAAACA
chIFN-*γ*R	TTTCACCTTCTTCACGCCATC
ch*β*-actinF	ACACGGTATTGTCACCAACT
ch*β*-actinR	TAACACCATCACCAGAGTCC

**Table 4:** Biological characteristics of the parental and recombinant viruses.

	rClone30	rClone30-VP2L-chGM-CSF	rClone30-VP2L
HA	2^9^	2^8^	2^9^

lgEID_50_/0.1mL	8	7.6	8
MDT	>144	>144	>144
ICPI	0	0	0

**Table 5:** Protection efficacy against IBDV (BC6/85 strain) challenge of each group in chickens.

Group	BF/BW ratio^a^	Histopathological BF lesion score^b^						Protection^c^	IBDV in BF^d^
		0	1	2	3	4	5		
rClone30-VP2L-chGM-CSF	1.693 ± 0.347	6	1	0	0	0	0	7/7 (100%)	0
rClone30-VP2L	1.491 ± 0.363	3	2	1	1	0	0	5/7 (71%)	2
IBDV-B87 vaccine	2.853 ± 1.269	2	2	1	1	1	0	4/7 (57%)	3
rClone30-chGM-CSF	1.063 ± 0.286	0	0	0	3	4	0	0/7 (0%)	7
rClone30	0.974 ± 0.0.278	0	0	0	1	5	1	0/7 (0%)	7
Negative control	0.894 ± 0.237	7	0	0	0	0	0	7/7 (100%)	0
Challenge control	1.745 ± 0.379	0	0	0	0	5	2	0/7 (0%)	7

(a) BF/BW ratios was calculated by bursal weight × 1000 then divided by body weight and presented as the mean ± standard deviation from each group. (b) Histological bursal lesion scores were given to each chicken based on the increasing severity of bursal atrophy (0: no lesion, 1: slight change, 2: scattered or partial bursal damage, 3: 50% or less follicle damage, 4: 51–75% follicle damage, and 5: 76–100% bursal damage). (c) Protection was defined by the number of chickens with histopathological bursal lesion score 0 and 1/the number of chickens in the group. (d) Detected by real-time PCR. Number of chickens positive for IBDV in BF tissue at 96 h postchallenge.

## Data Availability

All data generated or analyzed during this study are included within the article.
